# Elevated methane levels in small intestinal bacterial overgrowth suggests delayed small bowel and colonic transit

**DOI:** 10.1097/MD.0000000000010554

**Published:** 2018-05-25

**Authors:** Jaspreet Suri, Rahul Kataria, Zubair Malik, Henry P. Parkman, Ron Schey

**Affiliations:** a Gastroenterology Section, Department of Medicine; b Department of Medicine, Temple University School of Medicine, Philadelphia, PA, USA.

**Keywords:** colon, delay, hydrogen, methane, motility, SIBO, small intestine

## Abstract

Limited research exists regarding the relationship between small intestinal bacterial overgrowth (SIBO), small bowel transit (SBT), and colonic transit (CT). Furthermore, symptom analysis is limited between the subtypes of SIBO: hydrogen producing (H-SIBO) and methane producing (M-SIBO). The primary aims of this study are to: compare the SBT and CT in patients with a positive lactulose breath test (LBT) to those with a normal study; compare the SBT and CT among patients with H-SIBO or M-SIBO; compare the severity of symptoms in patients with a positive LBT to those with a normal study; compare the severity of symptoms among patients with H-SIBO or M-SIBO.

A retrospective review was performed for 89 patients who underwent a LBT and whole gut transit scintigraphy (WGTS) between 2014 and 2016. Seventy-eight patients were included. WGTS evaluated gastric emptying, SBT (normal ≥40% radiotracer bolus accumulated at the ileocecal valve at 6 hours), and CT (normal geometric center of colonic activity = 1.6–7.0 at 24 hours, 4.0–7.0 at 48 hours, 6.2–7.0 at 72 hours; elevated geometric center indicates increased transit). We also had patients complete a pretest symptom survey to evaluate nausea, bloating, constipation, diarrhea, belching, and flatulence.

A total of 78 patients (69 females, 9 males, mean age of 48 years, mean BMI of 25.9) were evaluated. Forty-seven patients had a positive LBT (H-SIBO 66%, M-SIBO 34%). Comparison of SBT among patients with a positive LBT to normal LBT revealed no significant difference (62.1% vs 58.6%, *P* = .63). The mean accumulated radiotracer was higher for H-SIBO compared to M-SIBO (71.5% vs 44.1%; *P* < .05). For CT, all SIBO patients had no significant difference in geometric centers of colonic activity at 24, 48, and 72 hours when compared to the normal group. When subtyping, H-SIBO had significantly higher geometric centers compared to the M-SIBO group at 24 hours (4.4 vs 3.1, *P* < .001), 48 hours (5.2 vs 3.8, *P* = .002), and at 72 hours (5.6 vs 4.3, *P* = .006). The symptom severity scores did not differ between the positive and normal LBT groups. A higher level of nausea was present in the H-SIBO group when compared to the M-SIBO group.

Overall, the presence of SIBO does not affect SBT or CT at 24, 48, and 72 hours. However, when analyzing the subtypes, M-SIBO has significantly more delayed SBT and CT when compared to H-SIBO. These results suggest the presence of delayed motility in patients with high methane levels on LBT.

## 1 Introduction

Gut microbiota can perform a variety of functions such as forming a barrier of defense, aiding in digestion of food, regulating electrolyte and water absorption, or helping to maintain overall homeostasis of the gastrointestinal tract. In small intestinal bacterial overgrowth (SIBO), this system becomes dysregulated through the altered or increased presence of bacteria in the small intestine. It has been associated with several conditions such as irritable bowel syndrome (IBS), inflammatory bowel disease (IBD), celiac disease, previous gastric and enteric surgery, cirrhosis of the liver, and chronic pancreatitis.^[1]^ SIBO results in symptoms such as flatulence, bloating, or diarrhea. The current gold standard in diagnosing SIBO entails jejunal aspirate samples taken endoscopically. Due to the invasive nature of obtaining jejunal aspirate samples, hydrogen breath testing-with substrates such as glucose or lactulose (LBT)-has become a widely accepted alternative.^[2]^ Several studies have evaluated the use of LBT in diagnosing SIBO; however, the results have not been conclusive due to several study limitations, including sample size and the discrepancy in cutoffs used for a positive test.^[3]^ Despite this, LBT has been adopted as the method of testing at many institutions due to the availability and ease of administration.

The metabolism of carbohydrates by the gut bacteria produces several byproducts, which are absorbed from the GI tract and ultimately, exhaled in the breath. These byproducts include carbon dioxide (CO_2_), hydrogen (H_2_), and methane (CH_4_). Lactulose is a nonabsorbable carbohydrate metabolized by bacteria in the colon leading to the production of hydrogen and/or methane gas and is one of the substrates (in addition to glucose, or d-xylose) that can be used to diagnose SIBO. After the ingestion of a carbohydrate substrate, the concentration of H_2_ and CH_4_ gas in parts per million (ppm) from the breath is measured over an hour.^[4]^ A great deal of controversy exists over the cutoffs used to define a positive hydrogen breath test; however, Erdogan et al^[5]^ compared glucose breath hydrogen testing with duodenal aspiration/culture and showed a cutoff value of ≥20 ppm hydrogen above baseline had a sensitivity and specificity of 77% and 66%, respectively.

Recently, there have been associations made between IBS-constipation type and elevated levels of methane on LBT when compared to diarrhea subtype or controls,^[6]^ as well as elevated methane production and delays in colonic transit (CT).^[7]^ Some of the proposed mechanisms include the initiation of a reflex pathway that starts when the distal small bowel is exposed to methane which produces slowing in the proximal intestinal segment, or the generation of nonpropagating small bowel contractions because of methane generation by intestinal bacteria.^[8]^ There has been limited data published on the effect of hydrogen-predominant SIBO (H-SIBO) compared to methane-predominant SIBO (M-SIBO). Furthermore, there is scarcity in data comparing the severity of symptoms of patients that may have methane SIBO (M-SIBO) versus strictly hydrogen producing SIBO. Whether the reported slowing of the intestinal content leaves patients with more nausea, constipation, gas, or diarrhea is unclear.

Thus far, intestinal motility has been assessed by substrate-hydrogen breath testing, radiopaque markers for CT, wireless motility capsule (WMC), and whole gut transit scintigraphy (WGTS). WGTS has shown to be a validated, relatively easy method of assessing complete intestinal motility. It utilizes food products typically eaten in the average diet to illustrate total and regional transit whereas using indigestible solid particles with radiopaque markers or a capsule may not accurately portray movement of food through the gastrointestinal tract due to its composition.

Hence, our aims in this study were to: compare SBT and CT between patients with a positive LBT and normal LBT using WGTS; compare SBT and CT between patients with H-SIBO to M-SIBO using WGTS; compare the severity of symptoms in patients with a positive LBT to those with a normal LBT; conduct subgroup analysis comparing the severity of symptoms between patients with H-SIBO and M-SIBO.

## 2 Methods

### 2.1 Subjects

This study was performed at Temple University Hospital, Department of Gastroenterology (Philadelphia, PA). It was a retrospective coded database study that did not involve direct patient contact and did not require patient consent, therefore no ethics committee review or IRB approval was needed. Data for all patients older than 18 years of age who completed a lactulose breath test and WGTS from January 2014 to July 2017 were reviewed. Measurements of H_2_ and CH_4_ in ppm, age, gender, and BMI, as well as symptoms recorded on the day of the LBT, were extracted. Exclusion criteria included patients whose WGTS test and LBT were more than 2 years apart, recent use of antibiotics (<4 weeks), and previous gastrointestinal surgery.

### 2.2 Study procedures

#### 2.2.1 Lactulose breath test

We utilized lactulose as the substrate for diagnosis of SIBO. Patients were given instructions for preparation before the test. They were instructed to avoid antibiotics for 4 weeks before the test, and probiotics, stool softeners, stool bulking agents, or laxatives 1 week before the test. The day before the test their diet was limited to plain white bread, plain white rice, plain white potatoes, bakes or broiled chicken/fish, water, and nonflavored black coffee or tea. Butter or margarine, carbonated beverages, beans, pasta, fiber cereals, sugar, and high fiber foods were not permitted. A 12 hour fast was performed before the test with instructions to brush their teeth 2 hours before the test. During the test, they were instructed to avoid gum, tobacco, breath mints, or candy. To keep CO_2_-production constant, physical activity was prohibited during the test and the subjects remained quietly seated.

A single baseline breath sample was collected before ingestion of 10 g of lactulose, dissolved in 200 mL of tap water. End expiration breath samples were collected using a 750 cm^3^ bag (Quintron, Milwaukee, WI) and analyzed for the concentration of H_2_ and CH_4_ using a gas chromatography analyzer (Quintron Microlyzer Self Correcting Model SC, Quintron). Subsequently, breath samples were collected every 15 minutes for a total of 3 hours.^[9]^ When the amount of exhaled gas during breath testing is quantified graphically, a double peak pattern with the first rise occurring before 90 minutes representing bacterial overgrowth in the small intestine, followed by a second rise representing metabolism by normal bacteria in the colon has been accepted as a positive breath test.^[10]^ Finally, if at any point during the test, the CH_4_ level ≥10 ppm, it has also been considered positive.^[11]^ Patients were also asked to fill out the Patient Assessment of Upper Gastrointestinal Disorders-Symptom Severity Index (PAGI-SYM) survey during the test to measure the degree of nausea, bloating, constipation, diarrhea, belching, and flatulence experienced during the test (Scale of 0–5, 5 being very severe).

#### 2.2.2 Whole gut transit scintigraphy

WGTS includes a measurement of gastric emptying, small bowel transit (SBT), and CT by having the patient ingest a radiolabeled meal consisting of a solid component and liquid component bound to isotopes of 2 different elements. The patient's abdomen is then prospectively imaged using nuclear imaging as detailed below.

After investigating for relevant food allergies, patients were prepped for this procedure by discontinuing medications that may affect motility 48 to 72 hours before the start of the procedure. These included opiates, laxatives, anticholinergics, and prokinetic agents. They were required to fast at least 8 hours the night before the first day. To prevent blood glucose abnormalities from affecting motility in diabetic patients, the level was checked just before administration of radiolabeled food bolus on the first day, then daily upon repeat imaging. The patient received their regularly scheduled dose of insulin unless modifications were needed. The test was not performed if the blood glucose level was more than 250 mg/dL. On the first day of the procedure, the patient was fed a standardized radiolabeled solid plus liquid meal. The solid portion delivered 0.5–1 millicuries (mCi) of a technetium (99m-Tc) through 120 g of cooked egg white, 2 slices of plain white or wheat bread, 30 g of strawberry jam and 120 mL of water. The liquid portion delivered 0.1–0.2 (mCi) of indium (111-In) mixed in 300 mL of water. Nonabsorbable isotopes of both elements were used as part of the protocol. Images of the abdomen were obtained in a 128 × 128 pixel matrix using a medium-energy collimator. Initial solid phase (containing 99m-Tc) gastric emptying images of 60 seconds (s) each were acquired in the anterior and posterior projections at hour (h) 0, 1, 2, 3, and 4 to calculate geometric mean of activity in the stomach and whole abdomen. Anterior and posterior whole abdomen images of the liquid phase (containing 111-In) were also taken for up to 4 hours. Both processes required separate energy settings on the collimator that corresponded to either the 99m-Tc or 111-In isotopes. Per protocol, to complete data retrieval for SBT, repeat imaging was conducted at hour 5 and hour 6 in both anterior and posterior projections for 60 seconds using only the energy setting for 111-In; as the 99m-Tc isotope containing solid phase is best used only for gastric emptying. The patients then resumed their normal diet and activity. They returned at 24, 48, and 72 hours after ingesting the meal for a single set of paired anterior and posterior whole abdomen images displaying 111-In activity to assess colon transit. Images were again obtained using a medium energy collimator with a 128 ×128 pixel matrix. A cobalt position marker was placed on the iliac crest to aid in anatomic referencing for all imaging. To complete processing, all images were corrected for radioisotope decay.^[12]^ The geometric center of decay corrected counts of the anterior and posterior images were used to reflect the amount retained at the three predetermined time intervals.

### 2.3 Study analysis

#### 2.3.1 Lactulose breath test

**Figure 1:**
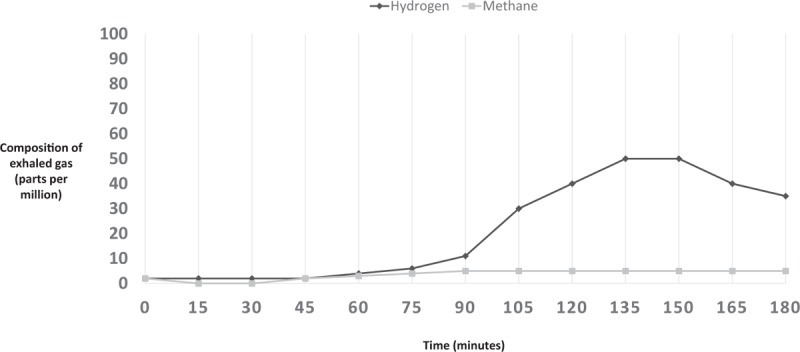
Representation of exhaled gas composition for a patient with a normal lactulose breath test.

**Figure 2:**
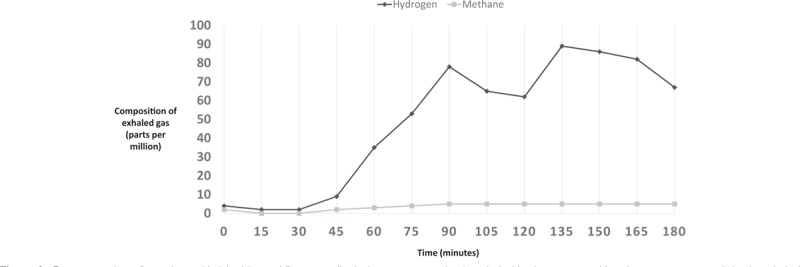
Representation of a patient with “double-peak” pattern displaying premature rise in exhaled hydrogen gas with subsequent expected rise in exhaled hydrogen later on in the time course of the LBT.

**Figure 3:**
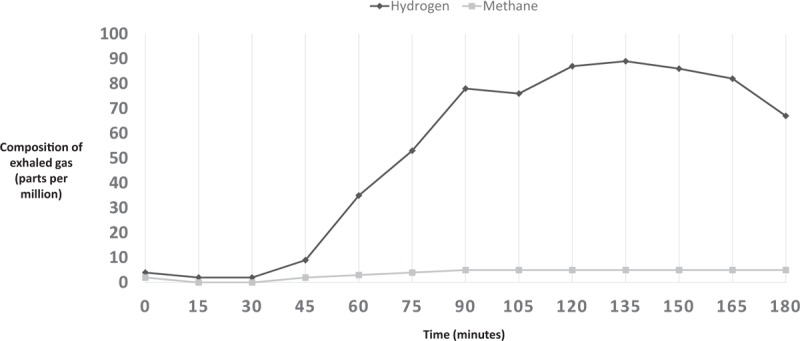
Representation of typical exhaled gas composition for a patient with hydrogen type SIBO.

**Figure 4:**
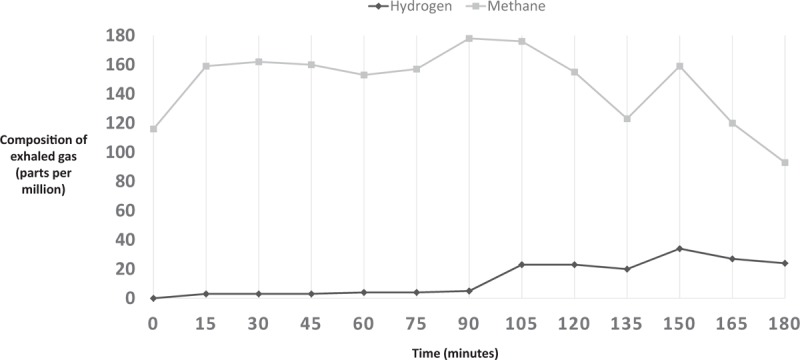
Typical exhaled pattern of gases for patient with methane type SIBO displaying rise in exhaled methane.

Breath H_2_ for each subject was analyzed to gather baseline value (in ppm) and peak values (in ppm). Results of a normal LBT are illustrated in Fig. 1. A LBT was considered positive for hydrogen-small intestinal bacterial overgrowth (H-SIBO) with a fasting breath hydrogen level of >20 ppm, the presence of a “double peak” (the first peak representing lactulose metabolism by the small intestinal bacteria and the second peak corresponding to lactulose reaching the cecum) (Fig. 2), or an early rise in breath hydrogen concentration of >20 ppm before 90 minutes (Fig. 3).^[5,6]^ For a LBT to be considered positive for M-SIBO, CH_4_ needed to be greater than or equal to 10 ppm at any point during the test (Fig. 4).^[7]^ If a LBT had evidence of both H-SIBO and M-SIBO, it was classified as M-SIBO.

#### 2.3.2 Whole gut transit scintigraphy

Due to the stasis that occurs when food reaches the terminal ileum reservoir, we were able to use the total amount of labeled 111-In accumulated at that juncture or transited into the cecum and ascending colon at 6 hours as an index of SBT.^[12]^ Normal SBT was defined as more than 40% of the radiolabeled food accumulated at the ileocecal region by 6 hours after ingestion.^[12]^ This was confirmed by visual interpretation of the images noting signs of residual activity in multiple loops of small bowel in relation to activity present at the ileocecal region. To measure CT, the geometric center of colonic activity was measured at 24, 48, and 72 hours. The geometric center is a weighted average of the radioactivity counted over specific segments of the colon. Each segment of the colon is assigned a number 1–7 starting from 1 being the cecum, then ascending colon, hepatic flexure, transverse colon, splenic flexure, descending colon, and finally rectosigmoid being number 7. The geometric center is calculated as the sum of a weighted fraction represented by the counts in each region multiplied by the region number divided by the total counts. A low geometric center means most of the radiolabeled material is near the cecum whereas a high geometric center means that most of it is in the rectosigmoid or has been excreted.^[12]^ Normal values at our institution, accounting for our equipment and protocol, were 1.6 to 7.0 at 24 hours, 4.0 to 7.0 at 48 hours, and 6.2 to 7.0 at 72 hours. All the data was extracted from the WGTS performed by our Nuclear Medicine department and organized in a spreadsheet for the purpose of comparison and analysis.

#### 2.3.3 Symptom severity assessment

We used the PAGI-SYM questionnaire administered at the time of the patient's LBT to assess severity of the symptoms they were experiencing. We focused on the most common complaints that patients with SIBO would usually present with: nausea, constipation, abdominal pain, diarrhea, belching, or flatulence. This was done for 2 levels of comparison; positive versus normal LBT and H-SIBO versus M-SIBO.

#### 2.3.4 Statistical analysis

All statistical analyses were performed using SAS 9.2. First, we evaluated the data for SBT. Using PROC GLM, we were able to assess for any degree of interaction between 2 variables. The data was evaluated to determine if the difference in the mean radiotracer accumulation at the IC valve at 6 hours between the normal LBT and positive LBT was significant; and then subsequently between the H-SIBO group and the M-SIBO group.

For CT, PROC MIXED model was used. We evaluated the differences in the means between groups at each time point (24, 48, and 72 hours). First, normal versus positive LBT groups were evaluated, then H-SIBO versus M-SIBO. All data were reported in means along with standard error of the mean.

## 3 Results

### 3.1 Patient characteristics

A total of 89 patients underwent both LBT and WGTS but only 78 were included as part of our analysis (Table 1). We excluded those with studies more than 2 years apart or those who had prior gastrointestinal surgeries, including intestinal resections, sleeve gastrectomy, gastric banding, or surgeries for bowel obstruction. We also excluded patients with incomplete studies including those who did not return at the appropriate time intervals for reimaging. The mean age was 48.0 ± 16.1 years and a mean body mass index of 25.9 ± 6.6. Thirty-one out of the 78 patients had a normal LBT. Of the remaining 47 patients with a positive LBT, 31 (66%) had H-SIBO, and 16 (34%) had M-SIBO.

**Table 1:**
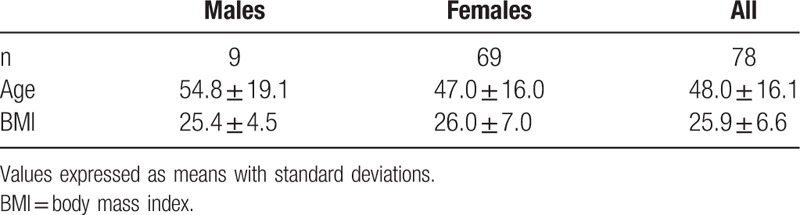
Demographics of patients included in our study.

### 3.2 Small bowel and colonic transit

When comparing SBT between patients with a normal LBT and positive LBT, those with a positive LBT had an average radiotracer accumulation of 62.1% at the ileocecal valve at 6 hours compared to 58.6% accumulation in the group with a normal LBT (*P* = .63). However, among patients with a positive LBT, those with H-SIBO had a mean accumulated radiotracer of 71.5%, compared to 44.1% in those with M-SIBO (*P* < .01) (Table 2).

**Table 2:**
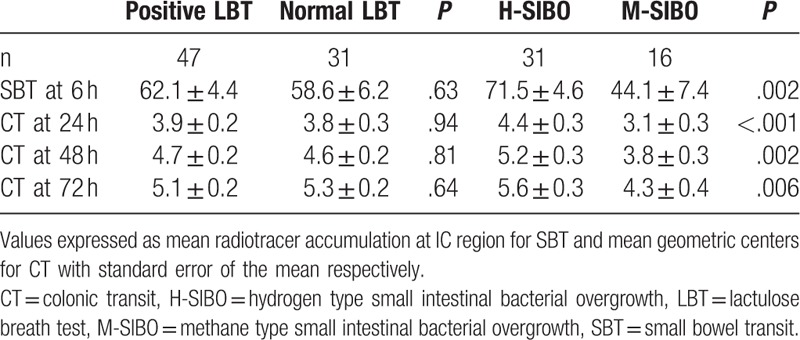
Values reflecting alteration of small bowel and colonic transit based on presence and type of SIBO.

For CT, the geometric centers of colonic activity were recorded at time intervals of 24, 48, and 72 hours. Comparing patients with a normal LBT to those with a positive LBT, the difference in CT was not statistically significant at all three time intervals (3.8 vs 3.9, 4.6 vs 4.7, and 5.3 vs 5.1, *P* = NS). However, among patients with a positive LBT, those with H-SIBO had significantly higher geometric centers compared to those with M-SIBO at each time interval (4.4 vs 3.1, 5.2 vs 3.8, 5.6 vs 4.3, *P* < .01) (Table 2).

### 3.3 Symptom assessment

No statistically significant difference was seen in nausea, bloating, constipation, diarrhea, belching, or flatulence between patients with a normal LBT versus those with a positive LBT. This was also true for the comparison of symptoms between H-SIBO versus M-SIBO. However, there was a significantly higher level of reported nausea amongst the H-SIBO group compared to the M-SIBO group (Table 3).

**Table 3:**
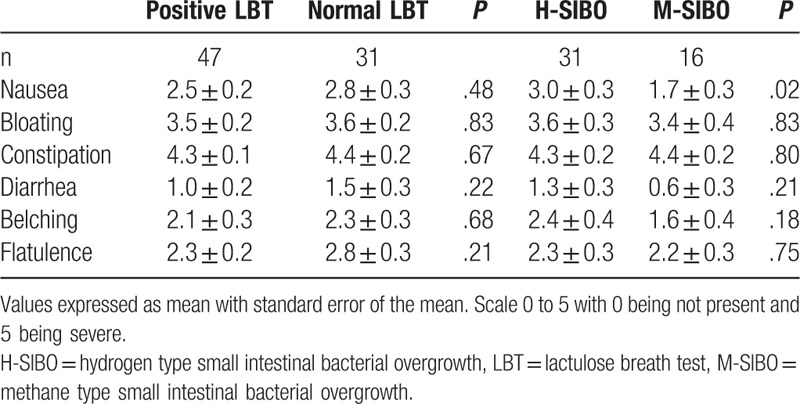
Symptom severity based on presence and type of SIBO.

## 4 Discussion

Our study reinforced the association between increased methane production in the gut and delayed small intestinal and colonic motility. This was demonstrated by the lower mean amount of radiotracer accumulation at the ileocecal valve at 6 hours and the lower mean geometric centers across all three time intervals for the M-SIBO group when compared to the H-SIBO group. This is in contrast to Rao et al who had demonstrated no significant relationship between methane gas and delay in small intestinal motility.^[13]^ We hypothesize that Rao et al provided contrasting results because they utilized the WMC in assessment of intestinal motility, a method that has shown to be less reliable in terms of segmental transit when compared to radioisotope studies.^[14]^ Our study is novel by evaluating correlation between WGTS and lactulose breath testing. Additionally, we provide a direct comparison between those qualified as hydrogen type SIBO versus those patients with significant levels of methane exhaled on breath testing (M-SIBO) to look at how severe this difference in intestinal delay actually is. Despite the fact that methane producers were associated with intestinal delay, there were no significant differences in their symptomatology aside from nausea, which was more severe in the hydrogen type SIBO group. Therefore, we believe that the symptom profile itself would be inadequate to differentiate between the 2 types and a formal breath test must be done.

Ongoing work is currently focused on the biochemical mechanism behind small intestinal and colonic delay caused by methanogens. Methane gas is produced in strictly anaerobic conditions by intestinal methanogens that metabolize hydrogen gas, a product itself of bacterial fermentation of sugar substrates. Currently the main culprits are thought to be single celled organisms belonging to the Kingdom Archaea including *Methaninobrevibacter smithii* and *Methanospaera stadmagnae* as well as bacteria species of the clostridium and bacteroides type.^[15]^ Hypotheses for the effect of methane gas on the intestinal physiology were discussed by Pimentel et al who showed that one explanation might be a feedback loop inhibiting contractile activity in the proximal intestinal segments when the distal segments gets exposed to excess amounts of methane or that methane may trigger repetitive, nonpropulsive contractions leading to delayed bolus transit times. In a separate study, it was also shown that methane producing IBS patients had lower postprandial serotonin levels in serum than the strictly hydrogen producing group.^[16]^ This is an important observation considering serotonin is the key mediator of the peristaltic reflex and its function is heavily related to the gastrointestinal tract. Regardless, the true mechanism by which methane gas causes adverse functionality of the human gastrointestinal system—and its associated symptoms—needs further investigation. As far as treatment is concerned, patients have noted improvement in symptoms with the current standard of care which includes 2 weeks of a nonabsorbable antibiotic such as Rifaximin for treatment of H-SIBO, or Rifaximin plus neomycin for the treatment of M-SIBO. Currently there are ongoing clinical trials into whether lovastatin, a commonly used drug to lower blood cholesterol levels, can curtail excess methane production by inhibiting the archaeal cell wall biosynthesis in animals and humans with IBS-constipation type.^[17]^

Our study is not without limitations. A larger sample size would provide us with potentially more significant differences in our end points as well as allow us to provide a more robust analysis to support our findings. Therefore, we may be more accurately able to explain why despite showing that methane type SIBO had lower amounts of radiotracer accumulation at the ileocecal region at 6 hours compared to hydrogen type, the comparison between positive and normal breath testing showed that small intestinal motility was actually faster in the positive group. Secondly, although we attempted to conduct both the WGTS and LBT within a few days apart if not on the same day, there were scheduling difficulties or barriers to doing so. Therefore, we could not establish a standardized time interval between the 2 tests. Nevertheless, this retrospective analysis provides insight on a growing cause for concern in gastrointestinal health.

In conclusion, small intestinal bacterial overgrowth, whether methane producing or strictly hydrogen, can have a significant effect on the patient's quality of life. Its symptoms are indeed real and can be controlled with our present interventions. Bloating and distention leading to abdominal pain, or nausea and constipation through changes in the neuromuscular signaling that regulates the gut, SIBO can be quite the nuisance. It can lead to malnutrition and weight loss if severe or vitamin and mineral deficiencies if milder. Some studies have related the occurrence of SIBO to age, while others have shown its propensity to develop in patients with other serious comorbidities as mentioned previously. The 2 processes most commonly predisposing healthy human individuals to SIBO are diminished gastric acid secretion and small intestinal dysmotility.^[18]^ Our study suggests that SIBO itself may be the cause of delayed bowel motility. Continued work on the mechanism behind its development or its persistence will aid in progress toward more targeted approaches for treatment in the future.

## Author contributions

**Jaspreet Suri:** study concept and design; data entry; analysis and interpretation of data; statistical analysis, drafting of manuscript.

**Rahul Kataria:** study design; analysis and interpretation of data; statistical analysis, critical revision of the manuscript for important intellectual content.

**Zubair Malik:** study concept and design, critical revision of the manuscript for important intellectual content.

**Henry P. Parkman:** study concept and design; analysis and interpretation of data; critical revision of the manuscript for important intellectual content.

**Ron Schey:** study concept and design; analysis and interpretation of data; study supervision, critical revision of the manuscript for important intellectual content.

**Conceptualization:** Jaspreet Suri, Rahul Kataria, Zubair Malik, Henry P. Parkman, Ron Schey.

**Data curation:** Jaspreet Suri, Rahul Kataria.

**Formal analysis:** Jaspreet Suri, Rahul Kataria, Henry P. Parkman, Ron Schey.

**Investigation:** Rahul Kataria, Zubair Malik, Henry P. Parkman, Ron Schey.

**Methodology:** Ron Schey.

**Supervision:** Ron Schey.

**Writing – original draft:** Jaspreet Suri.

**Writing – review & editing:** Rahul Kataria, Zubair Malik, Henry P. Parkman, Ron Schey.
